# Dislocation temporo-mandibulaire bilatérale survenue lors d'une éclampsie

**DOI:** 10.11604/pamj.2016.23.14.8270

**Published:** 2016-01-26

**Authors:** Nezha Oudghiri, Mouhssine Doumiri

**Affiliations:** 1 Service d'Anesthésie-Réanimation, Hôpital Maternité Souissi, Université Mohammed V, Rabat, Maroc

**Keywords:** Dislocation temporo-mandibulaire bilatérale, éclampsie, ATM, Bilateral temporo mandibular dislocation, eclampsia, TJM

## Image en medicine

La dislocation de l'articulation temporo-mandibulaire (ATM) est définie comme la perte permanente et plus ou moins complète des relations anatomiques normales entre les condyles mandibulaires et temporales. Les facteurs de risque prédisposant à ces dislocations sont connus et comprennent des conditions telles que le dimorphisme, le syndrome de l'appareil de la mastication algo-dysfonctionnel et les antécédents de la dislocation. Ces dislocations se produisent le plus souvent après un effort de bâillement ou en cas d'ouverture forcée volontaire de la bouche. Une prédominance féminine est retrouvée dans la littérature, et serait liée, d'abord à un relâchement du ligament ordinaire en raison de l'imprégnation hormonale et d'autre part à un traitement contraceptif oestroprogestatif qui augmenterait la probabilité d'un dysfonctionnement de l'ATM. Un cas de dislocation chez une parturiente criant de douleurs lors d'un accouchement voie basse a déjà été publié dans la littérature. Nous rapportons une circonstance rare d'occurrence de cette complication: une dislocation de l'ATM lors d'une crise d’éclampsie survenant à 32 SA. Ceci a été découvert en post extubation après une prise en charge appropriée de l’éclampsie césarisée sous anesthésie générale : l'examen n'a pas révélé d'histoire passée de dislocation de l'ATM mais une ouverture bloquée de la bouche, une impossibilité d'occlusion dentaire et une vacuité des deux glénoïdes temporales sur le scanner. La dislocation a été réduite manuellement, sous sédation par le propofol. Un bandage d'immobilisation de l'articulation a été mis en place pour quelques jours avec une bonne évolution clinique et fonctionnelle.

**Figure 1:**
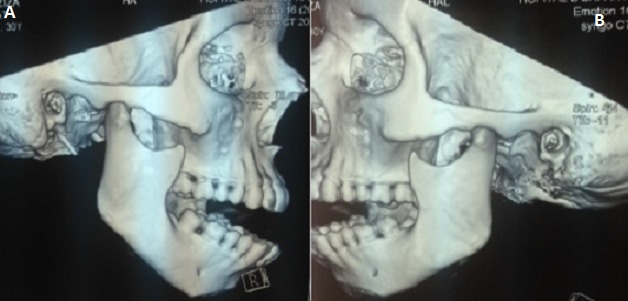
(A) image scannographique montrant une dislocation temporo mandibulaire droite; (B) image scannographique montrant une dislocation temporo mandibulaire gauche

